# Unmet Healthcare Needs among College Students during the COVID-19 Pandemic: Implications for System-Wide and Structural Changes for Service Delivery

**DOI:** 10.3390/healthcare10081360

**Published:** 2022-07-22

**Authors:** Monideepa B. Becerra, Thomas Charles Roland, Robert M. Avina, Benjamin J. Becerra

**Affiliations:** 1 Center for Health Equity, Department of Health Science & Human Ecology, California State University, San Bernardino, CA 92407, USA; rolat300@coyote.csusb.edu (T.C.R.); robert.avina@csusb.edu (R.M.A.); 2 Center for Health Equity, Department of Information and Decision Sciences, California State University, San Bernardino, CA 92407, USA; benjamin.becerra@csusb.edu

**Keywords:** mental health, sexual health, healthcare access, college students

## Abstract

Background: During the COVID-19 pandemic, college students faced health disparities in addition to a negative burden on academic performance; however, little is reported in the literature regarding healthcare utilization. Methods: A cross-sectional survey was conducted among consenting college student participants aged 18 or older from a Hispanic-serving institution. Descriptive and bivariate statistics were used to analyze demographic characteristics and the types of healthcare services needed by such characteristics. Logistic regression was used to adjust for noted sex differences in associations between reporting limited healthcare services and types of healthcare services. Results: The study population of 223 participants was mostly Hispanic/Latino (65%) and female (73%). Of the population, 11% reported they could not obtain needed healthcare services, with time being reported as the most common reason. Significant associations were found between seeking general healthcare services/routine screening, seeking mental health services, and seeking sexual health services with reporting limited healthcare services, with sex-adjusted odds ratios and 95% confidence intervals of 1.90 (95% CI: 1.08, 3.36), 3.21 (95% CI: 1.44, 4.15), and 2.58 (95% CI: 1.05, 6.35), respectively. Conclusions: Availability and inability to obtain health services may exacerbate college student health disparities. Targeted interventions are needed in the population to mitigate the potential burdens of unmet healthcare needs, particularly among minority college students.

## 1. Background

College students posit a unique population with their own patterns in health disparities. For example, the current body of literature highlights that college students are more likely to have a higher prevalence of poor mental health conditions but low utilization [1,2,3,4], as well as higher rates of sexually transmitted infections (STIs), coupled with risky sexual behavior and low sexual health literacy [5,6,7,8,9], among others. For example, results from the World Health Organization World Mental Health Surveys, representing 21 countries, demonstrate that one in five college students had a 12-month prevalence of mental health disorders, with approximately 16% reporting receipt of treatment for such disorders [1]. Likewise, a study assessing STI knowledge among college students found that less than 30% of participants scored 80% or higher [10], with similar patterns noted among racial/ethnic minority college students [11,12].

Furthermore, the COVID-19 pandemic has presented unique challenges for college students. For instance, a plethora of studies have highlighted the negative burdens on academic performance during the pandemic, including difficulty staying motivated and increased academic workload, among others [13,14,15]. For example, Osipov et al. reported that campus closures due to the pandemic significantly reduced physical activity as well as academic performance in physical exercise classes among the study participants, with a disproportionate burden shared among males [15]. Likewise, a study among Italian students noted that lowered concentration during learning as part of distance education, as well as anxiety related to the pandemic, etc., were related to poor academic outcomes of such students [16].

Furthermore, COVID-19 has also been shown to worsen health disparities among college students. For instance, in aiming to evaluate COVID-19-related mental health outcomes of college students, Son et al. [17] noted that a majority reported increased stress and anxiety, including fear/worry over the risk of infection, disruption of sleep patterns, etc., with similar patterns in worsened mental health and well-being noted by others as well [18,19]. For example, among college students in the United States, studies report worsened mental and physical health among the majority of study participants, as well as poor sleep health, including duration and wakefulness [20]. Likewise, stress related to academic outcomes was noted among college students, with a disproportionate burden among female students and students of color [21].

Cumulatively, while the literature highlights putative disparities during the pandemic, the limited assessment remains on patterns of healthcare utilization among college students during the pandemic. In this study, we addressed this gap in the literature and further aimed to assess healthcare utilization, types of services sought, and the availability of such services among primarily racial/ethnic minority college students.

## 2. Methods

Data were collected during the first quarter of 2022 from a federally designated Hispanic and minority-serving four-year public institution of higher education in the United States. During this phase, the majority of the United States was no longer under lockdown; however, the majority of courses were virtual. However, students were asked questions during the pandemic (thus the full year prior) and not just in 2022. All students enrolled on campus and aged 18 years or older were eligible to participate. Instructors of undergraduate and graduate classes, especially those teaching courses that had multiple majors, were asked to send the survey to their students with an incentive of extra credit. All data were collected via an anonymous online survey, and an unlinked page was used to collect the participants’ information for incentive purposes. Questions relevant to this study included the participants’ healthcare utilization, types of needed healthcare services, reported limitations on the availability of such services, as well as demographic characteristics of age, race/ethnicity, and sex at birth. We did not specifically differentiate between healthcare services being virtual (telemedicine versus not), as we sought to assess students’ unmet need for services, independent of the mode. Further, due to a lack of consistency across geographic areas on telemedicine, we could not ensure that the questions would be relevant to all participants.

Descriptive statistics were used to assess the prevalence and types of healthcare utilization and perception of the availability of services. Next, we used bivariate chi-square tests to assess if there were significant associations between the types of services and the reported availability of such services. Each outcome variable of interest was also assessed for associations with demographic characteristics. Those that yielded significant associations were then included in logistic regression analyses for adjusted models. All data were analyzed in SPSS version 28 (IBM, Corp.; Armonk, NY, USA), and an alpha value of 0.05 was used to denote significance. The present study was approved by the Institutional Review Board.

## 3. Results

Our study included 223 participants, all of whom were aged 18 years or older, currently enrolled at a four-year university, and consented to participate in the study. As shown in Table 1, the majority of study participants were aged 21–23 years (31.7%), closely followed by ages 27 years or older (30.8%). The highest prevalence of student self-identification of racial/ethnic group was Hispanic/Latino (65.4%). In addition, the majority of our population were females (73.1%).

As shown in Table 2, 66% of the participants reported that they went to a healthcare professional, medical center, or clinic for healthcare services, while 11% reported they needed healthcare services but could not obtain them. Of the types of healthcare services that were needed during the pandemic, the most common were COVID-19 testing (60.5%), COVID-19 vaccination (59.6%)**,** followed by general health/routine screening (48.9%), mental health services (19.3%), and sexual health services (14.3%). Furthermore, the most prevalent reason for citing an inability to obtain needed healthcare services was time. We did not find any statistically significant differences in healthcare utilization, including an inability to obtain care, by demographic characteristics (data not shown).

Next, we evaluated if a participant’s inability to obtain healthcare services differed by the type of services needed. As shown in Table 3, the results highlight that among those who needed general healthcare services/routine screening, a significantly higher percent reported not being able to obtain it compared to those who did not need such services (12.8% vs. 8.8%, p < 0.05). A similar pattern was noted for those seeking mental and sexual health services. On the other hand, among those who sought COVID-19 vaccination, a lower percent reported an inability to obtain care compared to those who did not seek such vaccination services (6% vs. 19.2%, p < 0.05). No significant associations were found between seeking other types of services and reporting limited availability (data not shown).

In addition, approximately 58% of the study participants reported that they felt the availability of healthcare services was limited due to the pandemic. When assessing if such reported limitations in the availability of healthcare services differed by demographic characteristics, we found a significant association with sex. For example, among females, as compared to males, a higher percentage reported that they felt healthcare services had been limited during the COVID-19 pandemic (63.2% vs. 46.4%, p < 0.05).

Further, as shown in Table 4, we found a significant association between the reported limited availability of healthcare services and the types of healthcare services that the participants needed during the pandemic. For example, a significantly higher percentage of participants who needed general health/routine screening felt that healthcare services were not available (67% vs. 49%, p < 0.05). Likewise, among those who needed mental health services, a significantly higher percentage reported that they felt the availability of healthcare services was limited compared to those who did not need such services (79.1% vs. 53.0%, p < 0.05). Among those seeking sexual health services, a higher percentage also reported limited availability of services compared to their counterparts (78.1% vs. 54.7%, p < 0.05). No significant associations were found between seeking other types of services and reporting limited availability (data not shown).

Due to sex differences in the reported availability of services, we conducted sex-adjusted logistic regression models to confirm if associations noted in bivariate analyses persisted. The results showed that those who sought general healthcare services/routine screening were also 90% more likely to report limited healthcare services (odds ratio [OR]: 1.90, 95% confidence interval [CI]: 1.08, 3.36) after adjusting for sex. Likewise, those seeking mental health services were also over three-fold more likely to report limited health services compared to those not seeking such services (OR: 3.21, 95% CI: 1.44, 4.15) in the sex-adjusted model. Finally, those who sought sexual health services were two-and-a-half-fold more likely to report limited healthcare services (OR: 2.58, 95% CI: 1.05, 6.35), even after adjusting for sex in the logistic regression model.

## 4. Discussion

The purpose of our study was to assess patterns in healthcare utilization, the types of services needed, and the availability of such services among college students during the COVID-19 pandemic. Our results demonstrated several key findings that provide insight into the need for targeted services for college students.

We found that the prevalence of reporting unmet healthcare needs (inability to obtain healthcare services often due to limited time and/or limited availability of healthcare services) was higher among those who needed general healthcare services/routine screening, mental health services, and sexual health services during the COVID-19 pandemic. Routine screening and healthcare check-ins are critical for ensuring long-term health and well-being, as well as the early identification and thus prevention of other chronic or severe illnesses [22,23]. The literature further notes that routine screenings are associated with optimal health outcomes and preventive care, including testing for cancers [24] and identifying previously unrecognized chronic illnesses, such as diabetes, pre-diabetes, and chronic kidney disease, among others [25]. Routine screenings are also a cost-effective means of preventing severe health outcomes [26,27,28].

Furthermore, the National Alliance on Mental Illness recommends routine screening for mental health among youth in order to ensure early detection [29]. Yet, research consistently demonstrates that college students have a disproportionate share of poor mental health conditions and associated risky behaviors [3,4,30,31], as well as low mental healthcare utilization [2,32]. Likewise, while sexually transmitted infections are prevalent among adolescents and young adults [33,34], the literature also notes limited sexual health literacy among college students [8,35], thus highlighting the importance of ensuring appropriate availability of such services to alleviate the low literacy among the population.

Cumulatively, the literature notes the importance of routine screening (general, mental, and sexual health) as well as the low utilization of services among college students. However, our results expand the empirical evidence by demonstrating that such disparities in the target population could be attributable to unmet healthcare needs in the form of limited availability and the inability to obtain such care, primarily due to time constraints in the target population. As such, tailored services for college students are needed.

For example, experts promote the need for flexible hours of operation [36,37], with after-hours services shown to significantly lower the odds of emergency department utilization [38,39]. Similar strategies can be integrated among college campuses to promote increased availability of healthcare services that do not routinely conflict with the student classes and/or work schedules. In turn, this may alleviate the burdens of unmet healthcare needed to be noted in our study.

This is especially critical given the importance of time constraints reported by our study participants as a primary factor for not being able to obtain needed care. Experts noted that electronic resources might provide an ideal scope of intervention in such situations [37,40,41]. Further, the literature shows that college students often rely on technology, including web searches, to obtain health information [42]. In addition, technology-driven health interventions have been shown to improve nutritional status [43], reduce weight gain [44], gain better stress management [45], and improve mental health-seeking scores [46] among college students. Given that a national assessment of sexual health services found lower availability among minority-serving institutions [36], integration of technology-based services that utilize less structural resources from institutions of higher education may be a putative scope of alleviating unmet healthcare needs of college students as noted in our study.

Furthermore, unlike the aforementioned patterns related to seeking general healthcare/routine services, mental healthcare services, and sexual healthcare services, our results show an opposite association among those seeking COVID-19 vaccination. For instance, those who sought vaccination for COVID-19 were less likely to report an inability to obtain care. This is likely due to the rigorous and synergistic efforts of public health and healthcare systems to make such services widely available to the public. For instance, coordinated efforts between the federal, state, local, and territorial governments (including a private sector commitment; a tiered strategy of delivery of such services; routine monitoring of supply chains; ongoing assessments of emergent disparities, incentives, and community-driven outreach programs; etc.) [47,48,49,50,51] have cumulatively led to the distribution and utilization of such needed care among the public. As such, these strategies are reflective of the best practices to provide coordinated interventions to combat other emergent issues among the most vulnerable, and thus can be adapted to delivering needed sexual and mental health screening services among the most at-risk.

The results of our study should be interpreted in the context of its limitations and strengths. The cross-sectional nature of the study design limits its ability to provide any causal relation; thus, further longitudinal analysis is needed to assess the duration of unmet healthcare needs during the pandemic. Given the sensitive nature of topics related to seeking mental and sexual healthcare services, our results may also be underreporting the true needs. However, we ensured the anonymity of our survey, thus aiming to reduce such a bias. It is also feasible that resource allocation and/or a shift from routine services, including mental and sexual health services, to that of COVID-19 management, may have several impacts. If that is putatively a factor, it further raises the importance of needed services and access to such services for the population. Notwithstanding the limitations, our study provides insight into the needed healthcare services predominantly among minority students during the COVID-19 pandemic. While the literature continues to highlight health disparities among college students, including mental and sexual health, our results highlight the putative burdens of unmet healthcare needs, both in terms of the availability and inability to obtain services, primarily due to time constraints. Such results highlight the need for system-wide and operational changes in healthcare delivery for such a disparity population, including the importance of telemedicine in ensuring adequate access to needed services.

## Figures and Tables

**Table 1:** Study population characteristics, n = 223.

Characteristic	Prevalence (%)
**Age**	
18–20 years	26.9
21–23 years	31.7
24–26 years	10.6
27 or more years	30.8
**Race/Ethnicity**	
Hispanic/Latino	65.4
Non-Hispanic minority	15.9
Non-Hispanic white	18.8
**Sex at birth**	
Female	73.1
Male	26.9

**Table 2:** Prevalence of healthcare utilization and types of services.

Variable	Prevalence (%)
**Healthcare service utilization**	
Needed and used services	65.9
Did not need services	23.2
Needed but could not obtain services	10.9
**Types of services**	
COVID-19 testing	60.5
COVID-19 vaccination	59.6
General health services/routine screening	48.9
Mental health services	19.3
Sexual health services	14.3
Other vaccinations	7.2
Other	9.9

**Table 3:** Inability to obtain healthcare services by types of services.

	Needed Services but Could Not Obtain It (%)
**Needed general health services/routine screening**	p < 0.001
Yes	12.8
No	8.8
**Needed mental health services**	p < 0.01
Yes	11.6
No	10.7
**Needed sexual health services**	p < 0.05
Yes	12.5
No	10.6
**COVID-19 vaccination**	p < 0.01
Yes	6.0
No	19.2

**Table 4:** Reported limited availability of healthcare services by service type.

	Reported Availability of Healthcare Services Were Limited (%)
**Needed general health services/routine screening**	p < 0.01
Yes	67.0
No	49.0
**Needed mental health services**	p < 0.01
Yes	79.1
No	53.0
**Needed sexual health services**	p < 0.05
Yes	78.1
No	54.7

## Data Availability

Not available for distribution per Institutional Review Board guidelines.
